# Allometric scaling for left ventricular mass and geometry in male and female athletes of mixed and endurance sports

**DOI:** 10.1186/s44156-024-00040-5

**Published:** 2024-02-14

**Authors:** David Oxborough, Danielle McDerment, Keith P. George, Christopher Johnson, Barbara Morrison, Gemma Parry-Williams, Efstathios Papatheodorou, Sanjay Sharma, Robert Cooper

**Affiliations:** 1 Research Institute for Sport and Exercise Sciences, Liverpool John Moores University, Tom Reilly Building, Byrom Street, Liverpool, L3 3AF UK; 2 Cardiology Department, Queen Elizabeth Hospital, University Hospitals Birmingham NHS Foundation Trust, Birmingham, UK; 3 School of Human Kinetics, Trinity Western University, Langley, BC Canada; 4 Cardiology Clinical and Academic Group, St George’s University of London, London, UK; 5 Onassis Cardiac Surgery Centre, Unit of Rare and Inherited Cardiac Disease, Athens, Greece

**Keywords:** Athlete, Echocardiography, Scaling, Left ventricular, Left ventricular mass

## Abstract

**Background:**

The athlete’s heart (AH) defines the phenotypical changes that occur in response to chronic exercise training. Echocardiographic assessment of the AH is used to calculate LV mass (LVM) and determine chamber geometry. This is, however, interpreted using standard linear (ratiometric) scaling to body surface area (BSA) whereas allometric scaling is now widely recommended. This study (1) determined whether ratiometric scaling of LVM to BSA (LVMi^ratio^) provides a size-independent index in young and veteran athletes of mixed and endurance sports (MES), and (2) calculated size-independent beta exponents for allometrically derived (LVMi^allo^) to BSA and (3) describes the physiological range of LVMi^allo^ and the classifications of LV geometry.

**Methods:**

1373 MES athletes consisting of young (< 35 years old) (males n = 699 and females n = 127) and veteran (> 35 years old) (males n = 327 and females n = 220) were included in the study. LVMi^ratio^ was calculated as per standard scaling and sex-specific LVMi^allo^ were derived from the population. Cut-offs were defined and geometry was classified according to the new exponents and relative wall thickness.

**Results:**

LVMi^ratio^ did not produce a size independent index. When tested across the age range the following indexes LVMi/BSA^0.7663^ and LVMi/BSA^0.52^, for males and females respectively, were size independent (r = 0.012; P = 0.7 and r = 0.003; P = 0.920). Physiological cut-offs for LVMi^allo^ were 135 g/(m^2^)^0.7663^ in male athletes and 121 g/(m^2^)^0.52^ in female athletes. Concentric remodelling / hypertrophy was present in 3% and 0% of young male and female athletes and 24% and 17% of veteran male and female athletes, respectively. Eccentric hypertrophy was observed in 8% and 6% of young male and female athletes and 9% and 11% of veteran male and female athletes, respectively.

**Conclusion:**

In a large cohort of young and veteran male and female MES athletes, LVMi^ratio^ to BSA is not size independent. Sex-specific LVMi^allo^ to BSA with LVMi/BSA^0.77^ and LVMi/BSA^0.52^ for male and female athletes respectively can be applied across the age-range. Population-based cut-offs of LVMi^allo^ provided a physiological range demonstrating a predominance for normal geometry in all athlete groups with a greater percentage of concentric remodelling/hypertrophy occurring in veteran male and female athletes.

## Background

The ‘Athletes Heart’ refers to the electrical, structural and functional cardiac changes that occur in response to chronic exercise training [1]. The magnitude and type of adaptation is dependent on factors including gender, ethnicity, sporting discipline, age, body size and type and training status [2]. Notable changes occur in the left ventricle (LV) with the greatest adaptation seen in athletes of high cardio-respiratory fitness i.e. those involved in mixed or endurance sports (MES) [3, 4]. Studies that have assessed young MES athletes (< 35 years old) compared to non-athletes, have demonstrated changes in LV geometry based on increased wall thicknesses and cavity size and concomitant elevation in LV mass (LVM) [5]. Although there is a predominance for normal geometry or eccentric hypertrophy [6], concentric remodelling/hypertrophy (increased wall thickness with normal cavity size) has been reported in up to 12% of young athletes [7].

Our interest in the veteran athlete (> 35 years old) has increased significantly due to older individuals having greater participation in structured MES alongside reported evidence of potential exercise-induced cardiac maladaptation [8]. That aside, the direct impact of life-long exercise training on LV geometry in the veteran athlete’s heart has not been fully reported. Moreso our understanding of the female heart in young and veteran athletes is less clear with few studies focusing on sex-differences of LV structure [9, 10] and although there is some evidence to suggest less adaptation in female compared to male MES athletes [7, 11] the nature and absolute magnitude of change still needs to be elucidated.

These proposed physiological adaptations can prove challenging for clinicians when an athlete undergoes pre-participation screening as these changes can often mirror pathological adaptation [12]. Echocardiography plays a fundamental role in the assessment of LV structure and function in the athlete and is specifically aimed at providing an estimation of chamber geometry. This is based on the calculation of LV mass indexed (LVMi) to body size alongside the calculation of relative wall thickness (RWT) [13]. Scaling of LVM to body surface area (BSA) is most commonly undertaken in a linear form, termed ratiometric scaling, in which the LVM is simply divided by BSA [14]. This form of scaling assumes there is a linear, proportional relationship between LVM and BSA, however in reality very few physiological variables are related to body size in this linear fashion [15] and therefore scaling of this type remains body size dependent. In fact, most biological relationships are allometric in nature and therefore based on this there is concern that LVMi using standard ratiometric scaling to BSA may provide inaccurate information, potentially providing false positive results causing anxiety and wasted resources in onward investigations, or false negative results that can inadvertently put an athlete at risk [16].

In view of this, it is pertinent to explore whether the current ratiometric methodology for deriving LVMi in a young and veteran MES athlete population is appropriate and if an allometric scaling approach is advantageous. In view of this, the aims of the current study are based on a large population of young and veteran male and female MES athletes and are three-fold, 1: to establish whether LVMi scaled ratiometrically to BSA (LVMi^ratio^) is independent of body size; 2: to determine size-independent (if appropriate age and sex specific) beta exponents for allometrically derived (LVMi^allo^) and 3: to determine the physiological range and subsequent classification of geometry.

## Methods

### Study population and design

One thousand three hundred and seventy three athletes aged 35 ± 17 (13 to 86) years from both mixed and endurance sports, provided written informed consent to participate in the study. This sample consisted of both males (n = 1026) and females (n = 347) of any ethnic background. Athletes were sub-divided into young (< 35 years old) (males n = 699 and females n = 127) and veteran (> 35 years old) (males n = 327 and females n = 220). For the purposes of this study an MES athlete was defined as an individual who was involved in competitive sport within a mixed or endurance discipline [5] of > 3 h structured exercise per week. Data were collected either as part of the mandatory pre-participation cardiac screening or as part of a structured research study, with athletes completing a health screening questionnaire to detail any cardiovascular symptoms, family history of SCD or other cardiovascular history as well as undertaking a resting 12-lead ECG and transthoracic echocardiogram. All results were reported by a sports cardiologist with clinical referrals made for any individuals who required further cardiac investigation and were subsequently excluded from the study. Ethics approval was obtained from the ethics committee of Liverpool John Moores University or St Georges University Hospital London.

### Procedures

#### Anthropometry

All participants underwent anthropometric assessment prior to cardiac screening. Body mass (Seca217, Hannover, Germany) and height (Seca Supra 719, Hannover, Germany) were recorded and body surface area (BSA) was calculated via the Mosteller equation, as previously described [17].

#### Echocardiography

A standard echocardiogram was performed by British Society of Echocardiography (BSE) accredited experienced sonographers using commercially available ultrasound systems (Vivid Q or Vivid E95, GE Healthcare, Horten, Norway) with a 1.5–4 MHz phased array transducer, with the participant lying in the left lateral decubitus position. All images were attained in accordance with the BSE guidelines [18]. Images were stored as a raw digital imaging and communications in medicine (DICOM) format and exported to an offline analysis system (EchoPac version 203, GE Healthcare, Horton, Norway) for subsequent analysis.

The parasternal long axis view was used to measure LV linear dimensions. The ventricular septum (IVSd), LV cavity (LVd) and posterior wall (PWd) were measured at end diastole from the 2D images and RWT was calculated using the formula {(IVSd + PWd)/LVd}. LV mass was ascertained using the ASE corrected equation (0.8 · 1.04 · {(IVS + LVID + PWT)^3^ − LVID^3^} + 0.6 g) [19]. LVMi was initially scaled ratiometrically to BSA (LVM^ratio^).

### Allometric scaling and statistical analysis

In order to assess the first aim, we plotted LVMi^ratio^ to BSA for all athletes and used a Pearsons Correlation to determine size independence. Where the correlation was significant, we proceeded to address the second aim of the study by establishing body size-independent indices for LVM. Here we adopted a sample-specific allometric exponent for the relationship between LVM and BSA using an iterative, nonlinear protocol of the order y = a:x^b. Once determined for the whole population we assessed age and sex as covariates within the model y = a:xb * exp(C * age) and y = a:xb * exp(C * sex) providing a coefficient C for each model. Where C was virtually 0 it was considered feasible to apply the same b exponent to all athletes across the age range or for males and females. If C was different to 0 we repeated the standard non-linear protocol to determine age or sex specific b exponents. Size independence for the new LVMi^allo^ was established in the same ways as described above i.e. for each group by running a Pearsons correlation of LVMi^allo^ to BSA.

Once size independent b exponents had been established we sought the final aim of the study by obtaining a definition of hypertrophy for allometrically scaled LVMi^allo^. The LVMi^ratio^ and LVMi^allo^ were plotted as x and y variables respectively for young and veteran males and females respectively. A polynomial equation was used to establish the relationship of the order y = mx^2^ + mx + c. The polynomial equation was then used to determine the LVMi^allo^ equivalent of LVMi^ratio^ at 115 g/m^2^ for males and 95 g/m^2^ for females [19]. Geometry was determined using this allometric scaled mass cut-off alongside the RWT of 0.42.

Absolute values were presented as mean ± SD with cut-off values for derived LVMi^allo^ defined as two standard deviations either side of the mean. Assessment of normal distribution was undertaken using a Kolomogorov Smirnov test and parameters that were normally distributed were compared using an independent t-test (non-parametric equivalent for non-normally distributed data) to establish differences between young and veteran and/or male and female athletes. A one-way samples ANOVA was used to establish differences between athlete demographics.

## Results

### Participant demographics

**Fig. 1:**
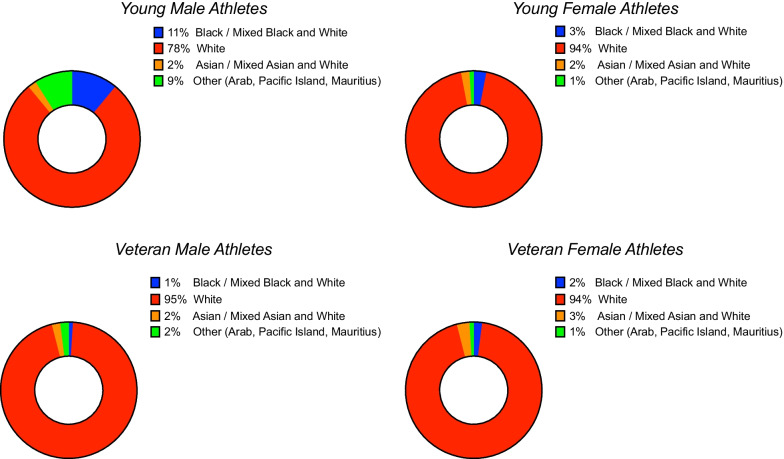
Athlete Ethnicity

Athlete demographics are presented in Table 1. As determined by selection, veteran athletes were significantly older than young athletes (P < 0.0001). There was no significant difference between the age of young males and females however female veteran athletes were significantly older than male veteran athletes (P = 0.0125). Young males were taller and heavier (P < 0.0001) than all athlete groups whilst veteran males were taller and heavier than all female athletes (P < 0.0001). Young female athletes were heavier than veteran female athletes (P = 0.031). Young male athletes had significantly greater training hours per week than veteran male athletes and young and veteran female athletes (P < 0.0001). Young female athletes had significantly higher training hours per week than both veteran male and female athletes (P < 0.0001). As expected, veteran athletes had significantly greater training duration years compared to young athletes (P < 0.0001) and female veteran athletes had greater training years compared to male veteran athletes (P = 0.024). All athlete groups were predominantly of white Caucasian ethnicity (see Fig. 1).

**Table 1:** Participant Demographics

Parameter	All athletes (n = 1373)	Young male athletes	Veteran male athletes	Young female athletes	Veteran female athletes
Age (years)	35 ± 17	22 ± 5	53 ± 8^^+^	22 ± 4	55 ± 7^*”#^
Height (cm)	176 ± 9	180 ± 7^º^”^	178 ± 7^+#^	166 ± 6	167 ± 6
Weight (kg)	74 ± 14	80 ± 14^º^”^	74 ± 9^+#^	64 ± 8*	60 ± 8
BSA (m^2^)	1.90 ± 0.21	2.0 ± 0.20^º^”^	1.91 ± 0.14^+#^	1.72 ± 0.12*	1.66 ± 0.12
Training (hrs per week)	14 ± 8	18 ± 8^^”º^	9 ± 3	14 ± 5*^+^	9 ± 3
Training Duration (years)	23 ± 15	14 ± 12	30 ± 14^^+^	13 ± 4	33 ± 12^*”#^

^*^Denotes significance < 0.05 between Young Females and Veteran Females

^Denotes significance < 0.05 between Young Males and Veteran Males

+Denotes significance < 0.05 between Young Females and Veteran Males

^”^Denotes significance < 0.05 between Young Males and Veteran Females

^º^Denotes significance < 0.05 between Young Males and Young Females

^#^Denotes significance < 0.05 between Veteran Males and Veteran Females

### Allometric scaling

**Fig. 2:**
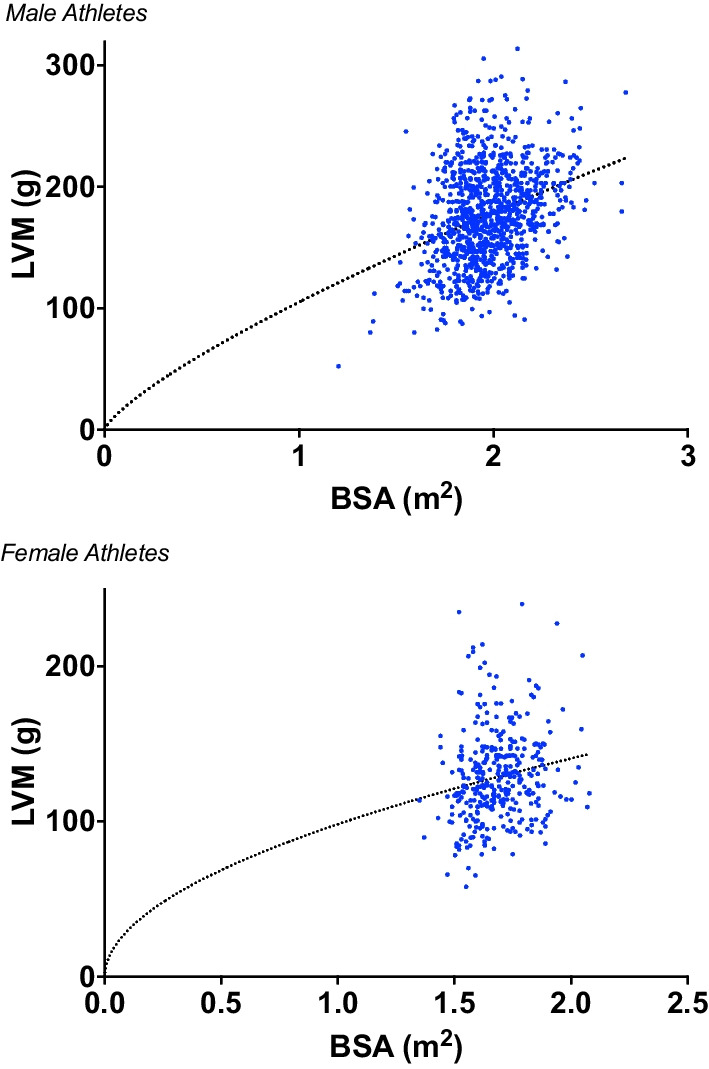
Non-linear regression of the order y = a:x^b for LVM and BSA for male and female athletes

Starting with the full athlete cohort and following ratiometric scaling of LVM to BSA (LVMi^ratio^) a significant correlation remained to BSA (r = 0.282; P = 0.0004) suggesting that size independence was not achieved with the standard approach. A non-linear b exponent of the order y = a:x^b was obtained for LVMi^allo^ (b = 1.135) which was size independent (r = 0.032; P =  > 0.05). Following testing with age and sex as co-variates the exponent was consistent across age range (C = 0.01) but not applicable to males and females (C = 0.202). Separate b exponents were therefore calculated for males (b = 0.7663) and females (b = 0.52) which when tested across the age range (LVMi/BSA^0.7663^ and LVMi/BSA^0.52^ for males and females respectively), were size independent (r = 0.012; P = 0.7 and r = 0.003; P = 0.920) (see Fig. 2).

Cut-off values for LVM, LVMi^allo^ and RWT are presented for young and veteran male and female athletes in Table 2. LVM and LVMi^allo^ were not significantly different between young and veteran males whilst RWT was significantly greater in veteran males (P < 0.0001). Absolute LVM was not different between young and veteran female athletes however indexed LVMi^allo^ and RWT were larger in veteran female athletes (P = 0.0006, P = 0071, P < 0.0001 respectively). RWT was significantly lower in young and veteran female athletes compared to their male counterparts (P < 0.0001 and P = 0.0096 respectively).

**Table 2:** Cut-off values for absolute LVM, allometrically scaled LVMi and RWT in young and veteran male and female athletes

Parameter	All male (n = 1026) Mean ± 2SD (lower–upper)	Young male (n = 699) Mean ± 2SD (lower–upper)	Veteran male (n = 327) Mean ± 2SD (lower–upper)	P value (young male vs veteran male)	All female (n = 347) mean ± 2SD (lower–upper)	Young female (n = 127) Mean ± 2SD (lower–upper)	Veteran female (n = 220) Mean ± 2SD (lower–upper)	P value (young female vs veteran female)
LVM (g)	176 ± 40 (96–256)	178 ± 38 (102–254)	174 ± 42 (90–258)	0.1498	129 ± 29 (70–187)	125 ± 23 (79–171)	131 ± 32 (67–195)	0.0699
LVMi^allo^ (g/(m^2^)^0.7663^)	105 ± 23 (59–151)	104 ± 21 (62–146)	106 ± 26 (54–158)	0.3088	–	–	–	–
LVMi^allo^ (g/(m^2^)^0.52^)	–	–	–	–	98 ± 22 (54–142)	94 ± 16 (62–126)	100 ± 25 (50–150)	0.0071
RWT	0.35 ± 0.06 (0.23–0.47)	0.33 ± 0.04 (0.25–0.41)	0.38 ± 0.07 (0.24–0.52)	< 0.0001*	0.34 ± 0.06 (0.22–0.46)	0.31 ± 0.04 (0.23–0.39)^^^	0.37 ± 0.06 (0.25–0.49)^#^	< 0.0001

^^^denotes P < 0.0001 males vs females

^#^denotes P = 0.0096 males vs females

### Left ventricular geometry

The polynomial relationship between LVMi^ratio^ and LVMi^allo^ was Y = − 0.0008^2^ + 1.3173x − 5.9165 and Y = − 0.0006x^2^ + 1.3436x − 1.14 for male and female athletes respectively. These equations allowed the equivalent cut-off for LVMi^ratio^ = 115 g/m^2^ of LVMi^allo^ = 135 g/(m^2^)^0.7663^) in male athletes and LVMi^ratio^ = 95 g/m^2^ of LVMi^allo^ = 121 g/(m^2^)^0.52^) in female athletes. The following geometry classifications were defined:

*Normal Geometry* male athletes LVMi^allo^ < 135 g/(m^2^)^0.7663^) and RWT ≤ 0.42; female athletes LVMi^allo^ < 121 g/(m^2^)^0.52^) and RWT ≤ 0.42, *Concentric Remodelling* male athletes LVMi^allo^ < 135 g/(m^2^)^0.7663^) and RWT > 0.42; female athletes LVMi^allo^ < 121 g/(m^2^)^0.52^) and RWT > 0.42, *Concentric Hypertrophy* male athletes LVMi^allo^ > 135 g/(m^2^)^0.7663^) and RWT > 0.42; female athletes LVMi^allo^ > 121 g/(m^2^)^0.52^) and RWT > 0.42 and *Eccentric Hypertrophy* male athletes LVMi^allo^ > 135 g/(m^2^)^0.7663^) and RWT ≤ 0.42; female athletes > 121 g/(m^2^)^0.52^) and RWT ≤ 0.42.

**Fig. 3:**
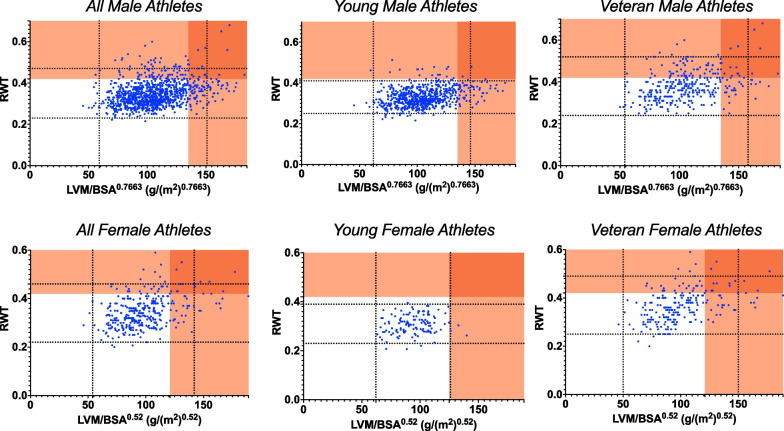
Allometrically scaled LVMI^allo^ and RWT demonstrating distribution of LV geometry. The dashed blue lines highlight the cut-offs for ± 2SD for both parameters and therefore the central box refers to the ‘physiological range’. The dark red shaded area indicates concentric hypertrophy whilst the orange shaded area above the RWT cut-off indicates concentric remodelling and the orange shaded area to the right of the LVMi^allo^ cut-off indicates eccentric hypertrophy

**Fig. 4:**
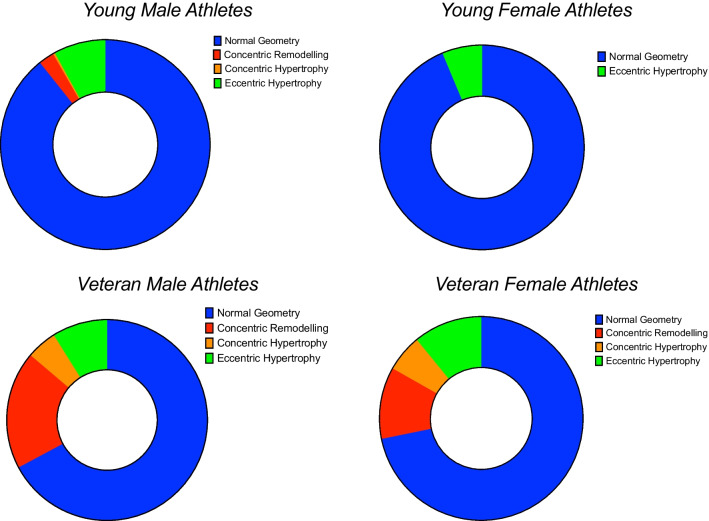
Classification of LV geometry

Figure 3 highlights the distribution of LV geometry for all, young and veteran male and female athletes. There was a lack of overlap of concentric remodelling or hypertrophy within the ‘physiological range’ for young athletes however it was present for veteran athletes with a significant proportion of the ‘physiological range’ being attributed to this type of geometry. Eccentric hypertrophy encompasses the ‘physiological range’ in young male athletes but not young female athletes whereas it was present in both male and female veteran athletes. The absolute percentages for specific geometry are presented in Table 3 and Fig. 4 highlighting the greater % of concentric remodelling and hypertrophy in the veteran athletes.

**Table 3:** Classification of LV geometry based on new LVM/BSA^0.7663^ and LVM/BSA^0.52^ criteria

Classification	All male athletes n (%)	Young male athletes n (%)	Veteran male athletes n (%)	All female athletes n (%)	Young female athletes n (%)	Veteran female athletes n (%)
Normal Geometry	844 (82.6)	624 (89.3)	220 (67.2)	277 (79.8)	119 (93.7)	158 (71.8)
Concentric Remodelling	79 (7.7)	17 (2.4)	62 (19.0)	25 (7.2)	0 (0)	25 (11.4)
Concentric Hypertrophy	18 (1.8)	2 (0.3)	16 (4.9)	13 (3.8)	0 (0)	13 (5.9)
Eccentric hypertrophy	85 (8.3)	56 (8.0)	29 (8.9)	32 (9.2)	8 (6.3)	24 (10.9)

## Discussion

To the best of our knowledge this is the first study to assess a sex specific size-independent index of LV geometry in a large cohort of young and veteran male and female MES athletes. The main findings of this study are (1) standard ratiometric scaling of LVM to BSA does not produce a size independent index in MES athletes, (2) sex-specific allometric scaling of LVM to BSA, LVMi/BSA^0.77^ and LVMi/BSA^0.52^, were size independent across the age-range in male and female MES athletes, respectively, and (3) age and sex-based physiological cut-offs demonstrate a predominance for normal geometry in all athlete groups with a greater percentage of concentric remodelling/hypertrophy occurring in veteran male and female athletes.

Seminal work by Morganroth [20] demonstrated a dichotomous nature of LV adaptation with concentric hypertrophy in young strength trained athletes and eccentric hypertrophy in young endurance athletes. The concentric limb of this hypothesis has since been refuted based on our developing knowledge of the physiological cardiac stimuli of acute and chronic exposure to exercise training and the mixed nature of sporting disciplines [21]. Our data support a predominance for normal geometry in young male and female MES athletes alongside a small proportion presenting with eccentric hypertrophy (8% of both female and male athletes) and concentric remodelling and hypertrophy (0% and 3% of female and male athletes respectively). Mixed and endurance sporting disciplines are defined by their relative contribution of isometric and isotonic activity with ‘mixed’ being related to alternate phases of dynamic and static workload and ‘endurance’ with a high degree of both dynamic and static activity [5]. These types of athletes represent those with the greatest degree of cardiac adaptation [4] and hence our findings of predominantly normal geometry are important when understanding the magnitude of adaption. A recent meta-analysis explored the impact of endurance training on LVM, demonstrating greater values compared to non-athletic controls [22]. These data provide further insight alongside the data presented here, that although LVM increases with athletic training, eccentric and more so concentric geometrical adaptation is relatively rare in young MES athletes.

Previous work is less clear regarding LV adaptation in older MES athletes primarily due to the multifactorial nature of adaptation including the impact of age alongside life-long training stimuli and variable/habitual lifestyle choices [23]. There are data however demonstrating greater cavity size and wall thicknesses in veteran athletes compared to controls [11]. Closer scrutiny of their data also highlights higher RWT in female athletes versus controls. Our findings support this and highlight the similar prevalence of eccentric LVH compared to younger athletes (8% and 11% in males and female older MES athletes respectively) but, importantly, more concentric hypertrophy/remodelling (24% and 17% in male and female MES athletes respectively). Concentric adaptation is a mechanism that occurs in response to an elevated afterload stimuli [24] and previous studies have highlighted an exaggerated blood pressure response to exercise as a major contributor to this type of adaptation [25]. Although we didn’t report blood pressure response to exercise in this study it could be speculated that a lifelong exposure to a subtle or marked hypertensive response during exercise could manifest later in an athlete’s career/life. Previous work has demonstrated that veteran MES athletes have superior diastolic function compared to non-active controls [26]. This may suggest that although there is concentric remodelling in these athletes, the nature of exercise training ensures that this adaptation remains physiological. Concentric remodelling as a natural ageing phenomenon in normotensive individuals has also been reported [27], and although greater concentric remodelling has been evidenced in veteran athletes compared to controls the sample sizes are relatively small [11]. It is apparent that larger longitudinal studies are required to determine the magnitude of concentric LV adaptation in older populations and specifically the impact of MES training on its development.

Echocardiography is invaluable in highlighting cardiac adaptation with measurements of LVM and RWT being fundamental in the assessment of athletic LV geometry [13]. The developed physiological ranges in this study are important to further direct the interpretation of this data and to support geometrical classifications. It is well established that scaling LVM for body size is important across populations [14] but more so in an athletic population in the presence of extremes of anthropometry and increased muscle mass. Our data highlights the need to develop physiological ranges based on an allometric approach and emphasises the limitations of standard ratiometric (linear) scaling. This is not a surprising finding based on the biological and scientific principles of geometric similarity [15] but it is at odds with current recommendations from echocardiographic professional bodies [28] where ratiometric scaling of LVM to BSA is advocated. Our findings are supported by other studies that have highlighted the importance of allometric scaling in athletes. Giraldeau et al. presented a collegiate athlete population and demonstrated that sex differences in absolute and ratiometrically scaled LVM were eliminated when allometrically scaled to lean body mass [29]. Whilst in a cohort of 464 junior athletes, it was found that size independence of LVM was achieved by scaling allometrically to BSA but with a different exponent (b = 1.5) than derived from our data [30]. This may be related to the wide-age range of our population, reflecting the interaction of both age and athletic adaptation on LVM. We also present different geometry between young and veteran athletes and therefore the different derived exponents likely encompass this structural variance. This further highlights the importance in deriving a *population* based exponent and although we found that a common exponent could be used across the age range it was not interchangeable between males and females. The exponents for male and female athletes were approximately 0.8 and 0.5 respectively and although the values are relatively similar, size independence was not achieved when using a common value. This has implications related to our physiological understanding of sex on the athlete’s heart suggesting that the association between LV remodelling and body size in an athletic population is different between males and females. It has been documented that the rate and magnitude of adaptation in LVM [22] and body composition [31] in response to endurance training differ between males and females. These differences have been related to both testosterone [32] and oestrogen levels with data on post-menopausal women being insightful highlighting increased LVM compared to pre-menopause [33]. In addition, it has been suggested that male athletes have a higher exercise related systolic blood pressure and therefore a potential subsequent impact on LV adaptation [34].

### Clinical implications

This study highlights the nature of LVM adaptation across the age-range in male and female MES athletes and the data informs us of an expected physiological range. This is important when interpreting echocardiography in the pre-participation screening environment when attempting to differentiate physiological from pathological adaptation and we advocate the equivalent cut-off for LVMi^ratio^ = 115 g/m^2^ of LVMi^allo^ = 135 g/(m^2^)^0.7663^) in male athletes and LVMi^ratio^ = 95 g/m^2^ of LVMi^allo^ = 121 g/(m^2^)^0.52^) in female athletes. The use of the allometric scaling exponents developed here refine the size independence of LVMi and improve the accuracy of the measurement when comparing across cohorts. A major challenge to clinical translation is often the cumbersome nature of employing an exponent and then being able to interpret the results. These data provide a starting point and a substrate for the use of advancing technology in large datasets as well as integrating web-based apps to facilitate their translation. It is also essential that these population-based exponents are reidentified and further tested to enable them to be applied in the research and clinical sports cardiology setting with confidence.

### Limitations

Although this is a large dataset we had fewer female athletes, a lack of pure resistance trained individuals and our population was not ethnically diverse. We would have liked to explore any derived exponents taking into account other demographics, however this was not feasible in this population but should be a consideration for future work. Echocardiographic assessment of LVM has inherent limitations and we acknowledge that the validity of this measurement is debateable when using it to characterise hypertrophy. The current study used equivalent LVMi^allo^ to determine geometry but as these were derived from the established ratiometric cut-offs the allocation of geometry is not different between approaches. That aside, These data highlight the distribution of geometry within the allometrically derived physiological range which would not have been possible had we provided ratiometric values. Further work is required to fully elucidate the extent of geometry using allometrically scaled LVMi and requires validation in future cohorts. As it is standard clinical practice to use LVMi derived geometry, it is therefore important to attempt to define athletic normality using this method.

## Conclusion

In conclusion, this study demonstrates that in a large cohort of young and veteran male and female MES athletes, standard ratiometric scaling of LVM to BSA does not produce a size independent index and we should consider applying sex-specific allometric scaling of LVM to BSA with LVMi/BSA^0.77^ and LVMi/BSA^0.52^ for male and female athletes respectively which can be applied across the age-range. Physiological equivalent cut-offs for LVMi^allo^ were produced and when using 135 g/(m^2^)^0.7663^ in male athletes and 121 g/(m^2^)^0.52^ in female athletes alongside standard values for RWT we demonstrate a predominance for normal geometry in all athlete groups with a greater percentage of concentric remodelling / hypertrophy occurring in veteran male and female athletes.

## Data Availability

The datasets generated and/or analysed during this study are not publicly available in order to protect privacy.

## References

[1] Pelliccia A, Maron BJ, Spataro A, Proschan M, Spirito P (1991). The upper limits of physiologic cardiac hypertrophy in highly trained elite athletes. N Engl J Med.

[2] Brown B, Somauroo J, Green DJ, Wilson M, Drezner J, George K (2017). The complex phenotype of the athletes heart: implications for pre-participation screening. Exerc Sport Sci Rev.

[3] D’Ascenzi F, Anselmi F, Piu P, Fiorentini C, Carbone SF, Volterrani L (2019). Cardiac magnetic resonance normal reference values of biventricular size and function in male athlete’s heart. JACC Cardiovasc Imaging.

[4] Pelliccia A, Culasso F, Di Paolo FM, Maron BJ (1999). Physiologic left ventricular cavity dilatation in elite athletes. Ann Intern Med.

[5] Pelliccia A, Caselli S, Sharma S, Basso C, Bax JJ, Corrado D (2018). European Association of Preventive Cardiology (EAPC) and European Association of Cardiovascular Imaging (EACVI) joint position statement: recommendations for the indication and interpretation of cardiovascular imaging in the evaluation of the athlete’s he. Eur Heart J.

[6] Utomi V, Oxborough D, Ashley E, Lord R, Fletcher S, Stembridge M (2014). Predominance of normal left ventricular geometry in the male “athlete’s heart”. Heart.

[7] Finocchiaro G, Dhutia H, D’Silva A, Malhotra A, Steriotis A, Millar L (2017). Effect of sex and sporting discipline on LV adaptation to exercise. JACC Cardiovasc Imaging.

[8] Eijsvogels TMH, Fernandez AB, Thompson PD (2016). Are there deleterious cardiac effects of acute and chronic endurance exercise?. Physiol Rev.

[9] Colombo CSSS, Finocchiaro G (2018). The female athlete’s heart: facts and fallacies. Curr Treat Options Cardiovasc Med.

[10] Kooreman Z, Giraldeau G, Finocchiaro G, Kobayashi Y, Wheeler M, Perez M (2019). Athletic remodeling in female college athletes: the “morganroth Hypothesis” revisited. Clin J Sport Med.

[11] Merghani A, Maestrini V, Rosmini S, Cox AT, Dhutia H, Bastiaenan R (2017). Prevalence of subclinical coronary artery disease in masters endurance athletes with a low atherosclerotic risk profile. Circulation.

[12] Martinez MW, Kim JH, Shah AB, Phelan D, Emery MS, Wasfy MM (2021). Exercise-induced cardiovascular adaptations and approach to exercise and cardiovascular disease: JACC State-of-the-Art Review. J Am Coll Cardiol.

[13] Oxborough D, Augustine D, Gati S, George K, Harkness A, Mathew T (2018). A guideline update for the practice of echocardiography in the cardiac screening of sports participants: a joint policy statement from the British Society of Echocardiography and Cardiac Risk in the Young. Echo Res Pr.

[14] Dewey FE, Rosenthal D, Murphy DJ, Froelicher VF, Ashley EA (2008). Does size matter? Clinical applications of scaling cardiac size and function for body size. Circulation.

[15] Tanner JM (1949). Fallacy of per-weight and per-surface area standards, and their relation to spurious correlation. J Appl Physiol.

[16] Batterham AM, George KP, Whyte G, Sharma S, McKenna W (1999). Scaling cardiac structural data by body dimensions: a review of theory, practice, and problems. Int J Sports Med.

[17] Mosteller R (1987). Simplified calculation of body surface area. N Engl J Med.

[18] Robinson S, Rana B, Oxborough D, Steeds R, Monaghan M, Stout M (2020). A practical guideline for performing a comprehensive transthoracic echocardiogram in adults: the British society of echocardiography minimum dataset. Echo Res Pract.

[19] Lang RM, Badano LP, Mor-Avi V, Afilalo J, Armstrong A, Ernande L (2015). Recommendations for cardiac chamber quantification by echocardiography in adults: an update from the American Society of Echocardiography and the European Association of Cardiovascular Imaging. J Am Soc Echocardiogr.

[20] Morganroth J, Maron BJ, Henry W, Epstein S (1975). Comparative left ventricular dimensions in trained athletes. Ann Intern Med.

[21] Haykowsky MJ, Samuel TJ, Nelson MD, La Gerche A (2018). Athlete’s heart: is the morganroth hypothesis obsolete?. Hear Lung Circ.

[22] Morrison BN, George K, Kreiter E, Dixon D, Rebello L, Massarotto RJ (2023). Effects of endurance exercise training on left ventricular structure in healthy adults : a systematic review and meta-analysis. Eur J Prev Cardiol.

[23] Parry-Williams G, Gati S, Sharma S (2021). The heart of the ageing endurance athlete: the role of chronic coronary stress. Eur Heart J.

[24] Lorell BH, Carabello BA (2000). Left ventricular hypertrophy: pathogenesis, detection and prognosis. J Am Coll Cardiol.

[25] Caselli S, Cicconetti M, Niederseer D, Schmied C, Attenhofer Jost C, Pelliccia A (2022). Left ventricular hypertrophy in athletes, a case-control analysis of interindividual variability. Int J Cardiol.

[26] Galetta F, Franzoni F, Femia FR, Bartolomucci F, Carpi A, Santoro G (2004). Left ventricular diastolic function and carotid artery wall in elderly athletes and sedentary controls. Biomed Pharmacother.

[27] Ganau A, Saba PS, Roman MJ, de Simone G, Realdi G, Devereux RB (1995). Ageing induces left ventricular concentric remodelling in normotensive subjects. J Hypertens.

[28] Harkness A, Ring L, Augustine DX, Oxborough D, Robinson S, Sharma V (2020). Normal reference intervals for cardiac dimensions and function for use in echocardiographic practice: a guideline from the British Society of Echocardiography. Echo Res Pract.

[29] Giraldeau G, Kobayashi Y, Finocchiaro G, Wheeler M, Perez M, Kuznetsova T (2015). Gender differences in ventricular remodeling and function in college athletes, insights from lean body mass scaling and deformation imaging. Am J Cardiol.

[30] George K, Sharma S, Batterham A, Whyte G, McKenna W (2000). Allometric analysis of the association between cardiac dimensions and body size variables in 464 junior athletes. Clin Sci.

[31] Ansdell P, Thomas K, Hicks KM, Hunter SK, Howatson G, Goodall S (2020). Physiological sex differences affect the integrative response to exercise: acute and chronic implications. Exp Physiol.

[32] Svartberg J, von Muhlen D, Schirmer H, Barrett-Connor E, Sundfjord J, Jorde R (2004). Association of endogenous testosterone with blood pressure and left ventricular mass in men. The Tromso Study Eur J Endocrinol.

[33] Subramanya V, Zhao D, Ouyang P, Lima JA, Vaidya D, Ndumele CE (2018). Sex hormone levels and change in left ventricular structure among men and post-menopausal women: the Multi-Ethnic Study of Atherosclerosis (MESA). Maturitas.

[34] Žemva A, Rogel P (2001). Gender differences in athlete’s heart: association with 24-h blood pressure: a study of pairs in sport dancing. Int J Cardiol.

